# INTERGENERATIONAL EMPLOYEE NETWORK AGEISM: COLLABORATIVE INTERVENTION TRAINING CREATES ATTITUDE MODIFICATIONS

**DOI:** 10.1093/geroni/igz038.2659

**Published:** 2019-11-08

**Authors:** Heath D Harllee, Amanda Noah, Becky P Knight

**Affiliations:** University of North Texas, Denton, Texas, United States

## Abstract

Lack of positive attitudes towards aging has shown to cause challenges within intergenerational networks in employment situations. These can include job satisfaction, intrinsic motivation to work, and ageism subjectivity as underlying determinants and consequences. The collaborative intervention pilot training program goals were two-fold: 1) To expose and understand ageism as a discriminatory action. 2) To create a more positive social dynamic network in a diverse workplace in regard to general expectations of ageism. Two team-based learning intervention programs were created in order to increase collaborative awareness of ageism and were presented to a medium size intergenerational department staff (N=64) as part of a professional development series on equity, diversity and inclusion. Through three multidimensional self-help training activities, learning was done individually, within similar age employee groups, and within intergenerational employee groups. Participants were able to discuss and express general understandings and expectations of aging and learning tools such as intergenerational reactivity and emotion regulation strategies were presented. Within survey responses at the completion of the trainings, key findings showed that respondents had a better understanding of ageism (76%) and felt better equipped to work within an employment team of diverse ages (71%). Additionally, the subject matter of this pilot training program resulted in re-conceptualized positive aging (61%). Future implications and goals for the program include interventions to further increase positive intergenerational understanding and workplace generational inclusiveness.

